# Anti-Inflammatory Effect of Dietary Pentadecanoic Fatty Acid Supplementation on Inflammatory Bowel Disease in SAMP1/YitFc Mice

**DOI:** 10.3390/nu16173031

**Published:** 2024-09-08

**Authors:** Drishtant Singh, Paola Mehghini, Alexander Rodriguez-Palacios, Luca Di Martino, Fabio Cominelli, Abigail Raffner Basson

**Affiliations:** 1 Department of Nutrition, School of Medicine, Case Western Reserve University, Cleveland, OH 44106, USA; dxs1087@case.edu; 2 Division of Gastroenterology & Liver Diseases, School of Medicine, Case Western Reserve University, Cleveland, OH 44106, USA; pxm295@case.edu (P.M.); axr503@case.edu (A.R.-P.); fxc90@case.edu (F.C.); 3 Digestive Health Research Institute, University Hospitals Cleveland Medical Center, Cleveland, OH 44106, USA; 4 Mouse Models Core, Silvio O’Conte Cleveland Digestive Diseases Research Core Center, Cleveland, OH 44106, USA; 5 Germ-Free and Gut Microbiome Core, Digestive Health Research Institute, Case Western Reserve University, Cleveland, OH 44106, USA; 6 Department of Molecular Biology and Microbiology, Case Western Reserve University, Cleveland, OH 44106, USA; 7 Case Digestive Health Research Institute, School of Medicine, Case Western Reserve University, Cleveland, OH 44106, USA; lxd150@case.edu; 8 Department of Medicine, School of Medicine, Case Western Reserve University, Cleveland, OH 44106, USA

**Keywords:** pentadecanoic acid, odd chain fatty acid, food supplementation, anti-inflammatory, inflammatory bowel disease

## Abstract

Background/Objectives: Dietary fats have been linked to the increasing incidence of chronic diseases, including inflammatory bowel diseases (IBD), namely, Crohn’s disease (CD). Methods: This study investigated the impact of pentadecanoic acid (C15:0), a type of an odd-numbered chain saturated fatty acid, for its potential anti-inflammatory properties in different mouse models of experimental IBD using the SAMP1/YitFc (SAMP) mouse line (14- or 24-week-old), including chronic ileitis and DSS-induced colitis. To quantitively assess the effect of C:15, we tested two dosages of C:15 in selected experiments in comparison to control mice. Intestinal inflammation and intestinal permeability were used as primary outcomes. Results: In ileitis, C:15 supplementation showed an anti-inflammatory effect in SAMP mice (e.g., a reduction in ileitis severity vs. control *p* < 0.0043), which was reproducible when mice were tested in the DSS model of colitis (e.g., reduced permeability vs. control *p* < 0.0006). Of relevance, even the short-term C:15 therapy prevented colitis in mice by maintaining body weight, decreasing inflammation, preserving gut integrity, and alleviating colitis signs. Conclusions: Collectively, the findings from both ileitis and colitis in SAMP mice indicate that C:15 may have therapeutic effects in the treatment of IBD (colitis in the short term). This promising effect has major translational potential for the alleviation of IBD in humans.

## 1. Introduction

Inflammatory bowel diseases (IBD) are a group of chronic gastrointestinal disorders, including Crohn’s disease (CD) and ulcerative colitis (UC), that have seen a significant increase in incidence over recent decades [1]. These disorders, characterized by persistent inflammation of the digestive tract, put a significant burden on the affected persons, impairing their quality of life and requiring regular medical attention and management [2]. UC affects only the colon and rectum, with continuous inflammation limited to the mucosal layer, while CD impacts any part of the gastrointestinal tract, with patchy, deep inflammation affecting the entire bowel wall [3]. Endoscopically, UC is identified by continuous small and superficial ulcers, while CD is characterized by deep fissuring ulcers and aphthous lesions. Histologically, the inflammation is limited to the mucosal layer in UC, whereas in CD, inflammation is transmural, affecting all layers of mucosa with granulomas as a distinctive feature [4]. Further, extraintestinal manifestations are commonly associated with both CD and UC. These manifestations might occur in the skin, eyes, joints, and biliary system, causing various other disease conditions like perianal disease, arthritis, pneumonitis, etc. [5].

IBD etiology involves a complex combination of genetic, environmental, and microbial variables [6]. As we obtain a better understanding of these factors, new research has revealed a potentially crucial role for dietary components, particularly dietary fats, in the development and management of IBD [7,8].

Fatty acids, as constituents of the human diet, serve as essential sources of energy and participate in a myriad of physiological processes within the body [9]. Essential constituents of dietary lipids, fatty acids contribute to the management of inflammatory bowel disease (IBD) through several mechanisms, including immune function regulation, gut microbiota modification, inflammation reduction, and preservation of the gut barrier [10,11]. Pentadecanoic acid (C15:0), an odd-chain saturated fatty acid, has garnered recent attention for its potential anti-inflammatory properties, especially within the context of inflammation-related diseases such as IBD [12,13,14]. While odd-chain fatty acids represent a minority of dietary fatty acids compared to their even-chain counterparts, they exhibit distinct biochemical and physiological characteristics [15]. C:15 is found in dairy products, particularly in the milk fat of ruminant animals such as cows [16], and has been used as a biomarker for dairy fat consumption [17].

Pentadecanoic acid has been proposed as an essential fatty acid because it is an established dietary fatty acid that is not readily produced by the body; has lower body levels associated with poorer cardiometabolic and liver health; and has demonstrated beneficial and pleiotropic activities directly related to cardiometabolic, immune, and liver health [18,19]. Extensive human cohort studies have demonstrated that higher pentadecanoic acid levels in the plasma are associated with lower long-term risks of chronic diseases such as type 2 diabetes, cardiovascular disease, and heart failure [20,21]. Furthermore, increased circulating pentadecanoic acid concentrations and higher dietary intake have been linked to lower mortality rates, increased lifespan, and lower risks of a variety of health disorders such as chronic inflammation, hypertension, and diabetes [22].

Despite these promising findings, the role of OCFAs, particularly C15:0, in IBD therapy is still unexplored. The aim of this work is to fill this gap by examining the effects of C15:0 supplementation in a mouse model of experimental colitis and chronic ileitis. We hypothesized that C15:0 exhibits anti-inflammatory characteristics that may provide therapeutic benefits in the treatment and prevention of IBD. In order to test this hypothesis, mice were given C15:0 at two physiologically relevant dosages as a diet supplement based on their hypothesized health benefits reported in earlier studies [14,18]. We focused on both the forms of IBD (i.e., UC and CD) to investigate if the anti-inflammatory benefits of C:15 can be effective across the varied inflammatory profiles shown in these different pathologies.

## 2. Materials and Methods

### 2.1. Animals and Measures to Improve Reproducibility

For this study, we used SAMP/YitFc mice (SAMP) raised under specific pathogen-free (SPF) conditions. Mice were provided by the Cleveland Digestive Diseases Research Center Mouse Models Core at Case Western Reserve University School of Medicine (CWRU). Mice were individually housed in germ-free NesTiso caging system on non-edible Aspen bedding, as previously described, to prevent bedding material ingestion as a confounding factor [23,24]. The mice were subjected to a 12 h light and 12 h dark cycle with their environment carefully controlled for temperature and humidity to ensure species-appropriate conditions in AAALAC-accredited Animal Research Center rooms. Several practical measures were taken to prevent artificial microbiome heterogeneity and to increase study power-driven reproducibility, as previously described [23,24]. The animals were euthanized utilizing carbon dioxide narcosis. All methods and studies were approved by the CWRU Institutional Animal Care and Use Committee to align with the principles outlined in the Guide for the Care and Use of Laboratory Animals (IACUC protocol number 2014-0158). All experiments were performed in compliance with criteria aimed at limiting variability in the microbiome and improving the study’s repeatability and statistical power [23,24]. For all experiments, to limit cage–cage microbiome variability, we performed inter-subject pre-experimental fecal homogenization (IsPreFeh) one week prior to the experiment commencement [25,26] Briefly, feces collected from each mouse were pooled together and homogenized in sterile PBS. The suspension was centrifuged at low speed to decant heavy particles and collect supernatant in a new tube. Similarly, the bedding material was soaked in PBS and mixed thoroughly to prepare a slurry, which was also centrifuged to collect supernatant. A combination of bedding and fecal supernatant (1:1 ratio) was used to gavage (~200–400 µL) the mice for 3 consecutive days to ensure fecal homogenization.

### 2.2. Spontaneous Ileitis and DSS Colitis in SAMP Mice

The study of the human IBD prototypes, CD, and UC has been facilitated in our ‘NIH-funded multi-institutional Cleveland Digestive Diseases Research Core Center’ with the use of the SAMP mouse, which is characterized by transmural inflammation and cobblestone lesions in the ileum of 100% of SAMP mice, which, by 14 weeks of age, resemble the pathological features of human CD. To facilitate the study of diets in intestinal inflammation simulating UC, the use of dextran sodium sulfate (DSS) to induce colitis is the most standard protocol across laboratories interested in IBD/UC. Herein, we used the SAMP mouse to test C:15 supplementation in mice without DSS (spontaneous ileitis) and with DSS (induced colitis). We focused on SAMP mice because no other model lines exist to resemble CD-like cobblestone ileitis [25].

### 2.3. Diet

The dietary pentadecanoic acid (powder form; Fatty15, Seraphina Therapeutics Inc., San Diego, CA, USA) was homogenized using 0.5% Sodium Carboxymethylcellulose (Merck, Rahway, NJ, USA) as a vehicle, and this homogenized suspension was used to gavage the mice orally. Initial experiments were intended to test of the amount of C:15 and were associated with a better anti-inflammatory effect using young, 14-week-old mice. The mice were kept on the standard mouse diet (CHOW P3000) before the start of the experiment, thus acting as the baseline diet, and were continued to access this diet till the end point of the experiment. To determine if there was a batch effect, the short-term feeding trials were repeated with a low number of animals twice to reach 6–7 mice/group.

### 2.4. Experimental Design

**Figure 1:**
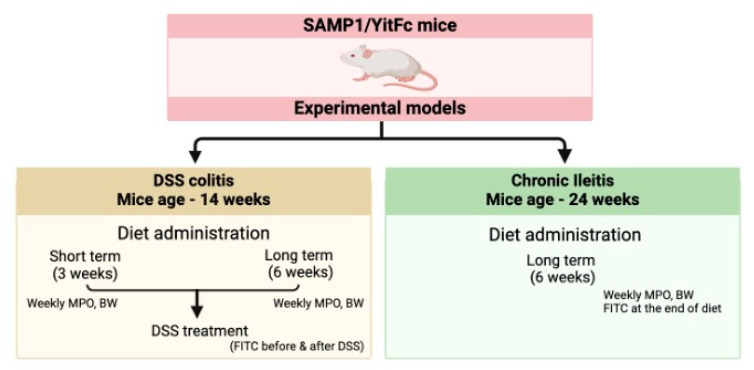
Overview of the Experimental Design (SAMP1/YitFc, a sub-strain of AKR/J mice produced through a program of selective breeding; DSS, Dextran Sulfate Sodium; MPO, myeloperoxidase; FITC, fluorescein isothiocyanate).

The feeding trials were aimed at assessing the anti-inflammatory properties of C:15 supplementation in ileitis and colitis in three (*n* = 3) context mouse models of experimental IBD using SAMP mice, as represented in Figure 1. In brief, feeding trials assessed the effect of C:15 on DSS-induced colitis (3%, 7 days) during a (i) 3-week-short and (ii) a 6-week-long-term C15 feeding DSS trial in 14-week-old SAMP (7 mice/group), and on (iii) SAMP chronic ileitis using 24-week old SAMP fed C15 for 6 weeks (6 mice/group). To quantitively assess if the effect of C:15 was dose-dependent, initial DSS experiments tested two dosages of 26 µg/mouse/day (herein referred to as 1X dose) and 52 µg/mouse/day (herein referred to as 2X dose) corresponding to volume equivalent to 10% of the mouse body weight (weight-to-volume), as previously recommended by our group for the oral administration of chemicals to laboratory mice [27]. The dosages were chosen based on the previous studies [14,15,18], and on the basis of recommended daily dose of humans, we calculated an equivalent dose for mouse according to average weight of mice and humans. Treatment groups were compared to mice receiving a daily dose of the control vehicle (0.5% Sodium Carboxymethylcellulose). Animals were gavaged daily in the mornings for 3 consecutive weeks.

### 2.5. DSS Induced Colitis and Colitis Severity Assessment

Mice in DSS studies were offered sterilized 3% (weight/volume) DSS dissolved in their drinking water for 7 days to induce colitis, which was followed by the discontinuation of DSS and supplementation of plain regular drinking water for 2 additional days. Mice were monitored daily for changes in body weight, the occurrence of fecal bleeding, the consistency of their stools, and their overall appearance to analyze the impact of colitis. Post-DSS, the inflammation in the colon was assessed via colonoscopy using a previously validated scoring method [28]. Isoflurane inhalation using an FDA-approved veterinary vaporizer (United States Pharmacopeia USP (Butler Schein Animal Health) was used to anesthetize mice prior to endoscopic procedures. No laxatives or fasting was implemented prior to colonoscopies. Mice were euthanized following the colonoscopy, and physical tissue characteristics such as colon length, body weight, and colitis severity were measured.

### 2.6. Analysis of Intestinal Inflammation and Gut Permeability In Vivo

Fecal samples were assessed weekly for quantification of fecal myeloperoxidase (MPO) activity and intestinal gut permeability using standard protocols before and after the DSS treatment. The fecal MPO activity tested is based on a previously described protocol using dianisidine-H_2_O_2_ adapted for use with 96-well plates [29]. Intestinal gut permeability in mice was evaluated before and after the DSS treatment using a method described previously [30]. In brief, the mice were subjected to overnight fast before being given a fluorescently labeled sugar probe, fluorescein isothiocyanate (FITC) Dextran (80 mg/mL in sterile PBS) via oral gavage. After 4 h, blood was collected in EDTA-coated vials, and plasma was appropriately diluted to analyze the fluorescence intensity using spectrometry (SpectraMax i3x, Molecular Devices, LLC., San Jose, CA, USA) that correlates with systemic FITC absorption and intestinal permeability.

### 2.7. Histopathological Analysis

The ilea and colons were collected and rinsed with sterile phosphate-buffered saline (PBS) after removal from the mice. Subsequently, the colon and ileal tissue were cut open longitudinally and preserved in 10% buffered formalin. The samples were washed with 70% ethanol after 24 h in fixative at 4 °C. The tissues were then embedded in paraffin and stained with Hematoxylin and Eosin (H&E). A blinded pathologist used a previously reported scoring system [25,31] to quantify intestinal inflammation, which included evaluating three essential parameters: (1) active inflammation, (2) chronic inflammation, and (3) villus architecture. Each of these factors was assigned a score ranging from 0 (normal tissue) to 3 (increasing levels of inflammation). All the samples in each group were assessed for quantification of intestinal inflammation.

### 2.8. nCounter Nanostring Gene Expression Analysis

RNA was isolated from the colon tissue and stored in RNA later using RNeasy Mini kit (Qiagen, Hilden, Germany) while following the manufacturer’s instructions. The RNA was assessed for quality and quantity using Nanodrop (Thermo Fisher, Waltham, MA, USA). The Nanostring core at Cleveland Clinic Foundation (CCF) carried out the gene expression analysis using Immune exhaustion panel (XT Mm Exhaustion CSO, Nanostring, Seattle, WA, USA) following the instructions of manufacturer.

### 2.9. Data Analysis

The data are expressed as a mean ± standard deviation (SD) using GraphPad Prism (version 10.3.1(464)). The parametric data were analyzed using ordinary one-way ANOVA and unpaired Student’s *t*-tests, while nonparametric data were assessed using Kruskal–Wallis and Mann–Whitney tests. Differences with p values less than 0.05 were considered statistically significant.

## 3. Results

### 3.1. Three-Week C:15 Supplementation Prevented the Occurrence of Severe DSS-Colitis in SAMP Mice

To determine if the consumption of C:15 could prevent the occurrence of severe colitis in a DSS colitis SAMP model, 14-week-old SPF-SAMP mice were randomly assigned to one of three groups to receive C:15 at 26 µg/mouse/day or 2X (52 µg/mouse/day) dosages or the control vehicle for a three-week period prior to the exposure of mice to DSS in the drinking water. At the end of a 3 wk feeding trial, DSS-treated SAMP mice given C:15 prior to DSS at either 1X (26 µg/mouse/day) or 2X (52 µg/mouse/day) dosages exhibited reduced severity of DSS colitis (body weight, colon length, histology, fecal MPO; Figure S1), with lower colonoscopy mean scores by the end of the 7-day DSS-treatment cycle [C:15(1X): 1.6 ± 0.8, C:15(2X): 1.6 ± 0.8 vs. control: 4.7 ± 0.9, ANOVA P = 0.03], as well as had improved gut permeability (i.e., less translocation of FITC-dextran from the gut lumen into the plasma), compared with controls (Figure S1). The data presented in Table 1 represents a summary of the results obtained in different experimental models in this study.

**Figure 2:**
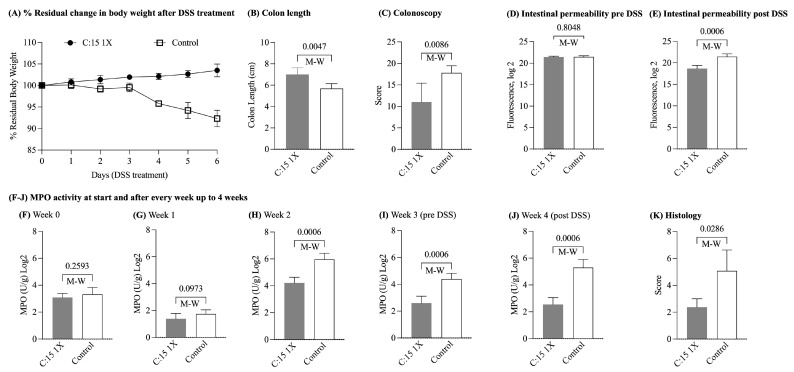
C:15 given for 3 weeks reduces the severity of acute chemical colitis in 14 wk old SPF SAMP. Values are mean ± SD, *n* = 7. (**A**) Effect of C:15 diet on body weight of DSS-treated mice, (**B**) colon length, (**C**) colonoscopy score, (**D**) intestinal permeability assay—FITC-Dextran before DSS treatment, and (**E**) intestinal permeability after DSS treatment; (**F**) fecal MPO activity before the start of diet, and after (**G**) 1 week and (**H**) 2 weeks of diet administration; (**I**) fecal MPO activity before DSS and (**J**) after DSS treatment; (**K**) colon histology scores (FITC, fluorescein isothiocyanate; MPO, myeloperoxidase; M-W, Mann–Whitney *U* test).

Based on the findings from this pilot study, we then repeated the experiment, increasing the N and focusing on the 1X dose (vs. control, n = 7/group). Significant alleviation of colitis symptoms was again observed in the mice treated with C:15 as compared to the control group (Figure 2). Compared to the control group, C:15 aided in maintaining the body weight of mice after DSS treatment (% Residual weight on the day of sacrifice; C:15(1X) 103.1 ± 2.0 vs. control: 92.0 ± 2.8, *t*-test *p* = 0.005; Figure 2A). The mice treated with C:15 had significantly longer colons (average colon length; C:15: 7.0 ± 0.6 vs. control: 5.6 ± 0.4, *p* < 0.0047; Figure 2B) and lower colonoscopy scores (mean scores; C:15: 3.1 ± 1.0 vs. control: 4.4 ± 1.5, *p* < 0.008; Figure 2C) compared to the control mice. Additionally, the FITC-dextran assay revealed the potential for C:15 to maintain intestinal integrity, evident by lower plasma fluorescence in the C:15 treated groups post-DSS treatment compared to the control mice (FITC Dextran Log_2_; C:15 18.6 ± 0.7, control: 21.4 ± 0.6, *p* < 0.001; Figure 2D,E). Further, C:15 exhibited anti-inflammatory properties, with treated mice exhibiting lower fecal MPO activity compared to the control group, both before and after DSS treatment (Figure 2F–J). The alleviation of colitis symptoms in the C:15 treated mice was also confirmed by lower colon histology scores (C:15: 11.0 ± 4.3 vs. control: 17.8 ± 1.6, *p* < 0.02) compared to control mice (Figure 2K). We then tested whether increasing the duration of C:15 supplementation would have a beneficial effect on DSS colitis.

### 3.2. Six-Week C:15 Supplementation Reduced the Endoscopic Severity of DSS-Colitis in SAMP Mice

**Figure 3:**
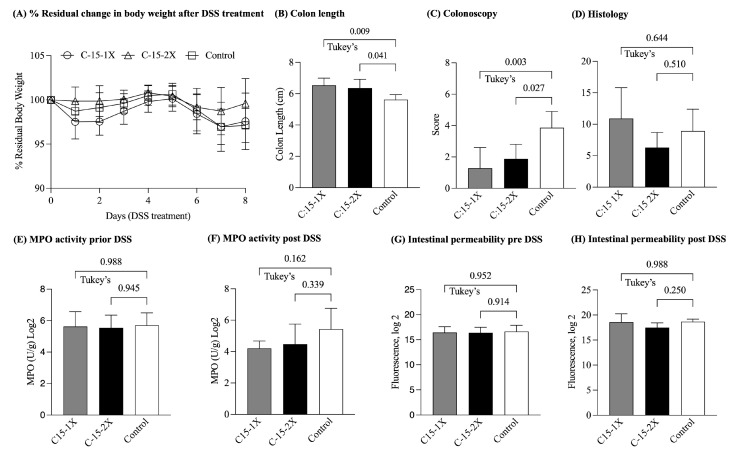
Effect of six weeks of C:15 supplementation on SPF-SAMP mice DSS colitis. Values are mean ± SD, *n* = 6. (**A**) Percentage change from original body weight (defined as day 0 and as 100%) after induction of DSS-colitis, (**B)** colon length, (**C**) colonoscopy score, (**D**) colon histology score, (**E**) fecal MPO activity before DSS and (**F**) after DSS treatment, and (**G**) FITC-Dextran before DSS and (**H**) after DSS treatment (FITC, fluorescein isothiocyanate; MPO, myeloperoxidase; KW—Kruskal–Wallis ANOVA, ANOVA—Analysis of Variance).

To determine if the consumption of C:15 for longer than 3 weeks could prevent or attenuate the occurrence of colitis of DSS-induced colitis, 14-week-old SPF-SAMP mice were randomly assigned to one of three groups to receive C:15 at 1X (26 µg/mouse/day) or 2X (52 µg/mouse/day) dosages or the control vehicle for a six-week period prior to the exposure of mice to DSS in the drinking water (Figure 3). The 6 wk supplementation, however, did not have a significant effect on body weight, fecal MPO, or intestinal permeability. C:15 demonstrated a positive impact on maintaining mouse body weight. However, there was not a significant difference between the groups (% residual weight on the day of sacrifice; C:15(1X): 97.5 ± 2.9, C:15(2X): 99.5 ± 2.5 vs. control: 97.1 ± 1.7, ANOVA, *p* = 0.2; Figure 3A). At the end of the 6 wk feeding trial, DSS-treated SAMP mice given C:15 exhibited greater colon lengths (average; C:15(1X): 6.5 ± 0.4, C:15(2X): 6.3 ± 0.5 vs. control: 5.6 ± 0.3, ANOVA, *p* = 0.008; Figure 3B) and lower post-DSS colonoscopy scores (mean scores; C:15(1X): 1.6 ± 0.8, C:15(2X): 1.6 ± 0.8 vs. control: 4.7 ± 0.9, ANOVA, *p* = 0.003; Figure 3C) compared to the control group. However, there was no significant difference in the colon histology scores between the C:15-treated and control groups (mean scores; C:15(1X): 10.9 ± 4.8, C:15(2X): 6.3 ± 2.3 vs. control: 8.9 ± 3.4, ANOVA, *p* = 0.1; Figure 3D). Additionally, there was no significant difference in the fecal MPO activity among various groups, indicating that long-term treatment of C:15 did not effectively reduce inflammation (fecal MPO activity Log_2_; C:15(1X): 3.1 ± 0.2, C:15(2X): 3.2 ± 0.9 vs. control: 7.1 ± 0.1, ANOVA, *p* = 0.1) (Figure 3E,F). Furthermore, based on the FITC-dextran assay results, it appeared that prolonged administration of C:15 did not protect against DSS-induced increased gut permeability (FITC dextran Log_2_; C:15(1X): 19.0 ± 2.1, C:15(2X): 17.6 ± 0.5 vs. control: 24.0 ± 0.5, ANOVA, *p* = 0.1) (Figure 3G,H). Overall, the findings suggest that the anti-inflammatory potential of the C:15 diet could be achieved within 3 weeks.

### 3.3. Six-Week Supplementation Reduces the Severity of Advanced Ileitis in SAMP Mice

**Figure 4:**
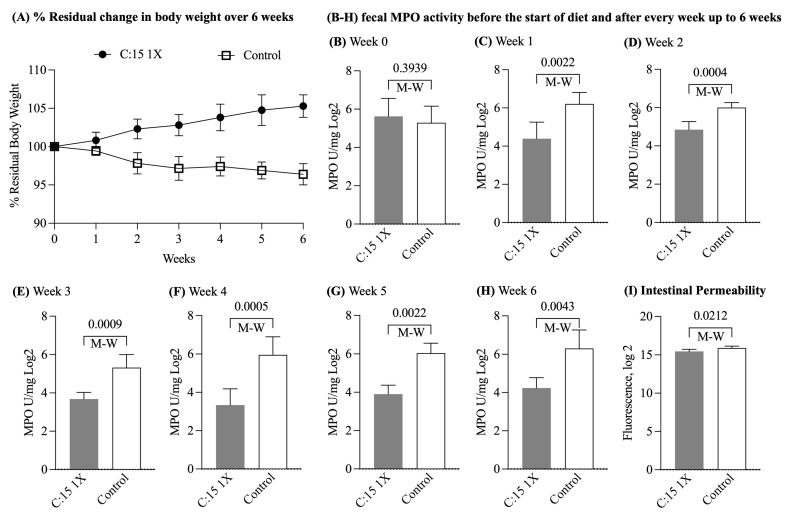
Treatment of older SPF-SAMP mice with C:15 for six weeks reduces the severity of established ileitis. Values are mean ± SD, *n* = 6. (**A**) Effect of C:15 diet on body weight over six weeks (percentage change from original body weight defined as week 0 and as 100% after the start of diet). (**B**) Fecal MPO activity before the start of diet. (**C**) Fecal MPO activity after 1 week, (**D**) 2 weeks, (**E**) 3 weeks, (**F**) 4 weeks, (**G**) 5 weeks, and (**H**) 6 weeks of diet administration. (**I**) Intestinal permeability assay—FITC-Dextran before sacrifice (FITC, fluorescein isothiocyanate; MPO, myeloperoxidase; M-W, Mann–Whitney *U* test).

We then sought to test the anti-inflammatory potential of C:15 in a model of established chronic ileitis (i.e., 24-week-old SAMP mice). After 6 weeks, the C:15-treated mice exhibited higher body weight (Figure 4A) and had significantly lower fecal MPO activity compared to the control group (Figure 4B–H). C:15-treated mice also had less translocation of FITC-Dextran (FITC Dextran Log_2_; C:15: 15.4 ± 0.2 vs. control: 15.8 ± 0.2, *p* = 0.02; Figure 4I) compared to non-treated mice, suggesting a protective role of C:15 on intestinal permeability and disease severity in older SAMP with established ileitis.

### 3.4. Three-Week C:15 Supplementation Altered the Expression of Various Genes Involved in Different Immunological Pathways in SAMP Mice

**Figure 5:**
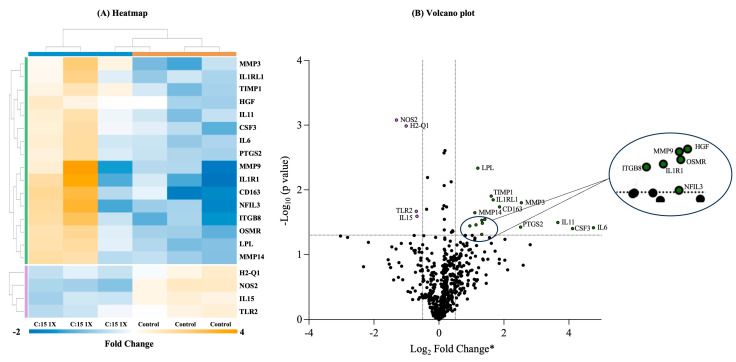
C:15 supplementation alters expression of various genes related to different immunological pathways in SAMP mice. (**A**) Heatmap and (**B**) volcano plot of the normalized data revealed connections among genes with differential expression * in the colon tissues of DSS-treated mice upon C:15 supplementation for 3 weeks in comparison to control. Each row in the heatmap represents a specific probe, and each column represents a specific sample (green bar represents upregulated gene clusters, and purple bar represents downregulated gene clusters). * Criteria for genes with differential expression in volcano plot were fold-change > ±1.5 and *p* < 0.05.

An immune exhaustion panel (XT Mm Exhaustion CSO, Nanostring, Seattle, WA, USA) was used to analyze the gene expression that targets ~785 genes associated with different immunological pathways. The data were analyzed using the online platform ROSALIND (https://nanostring.rosalind.bio, accessed on 24 July 2024), and heatmaps and volcano plots (Figure 5) were generated to visualize the clustering of genes that exhibited differential expression between the experimental groups. The genes were clustered according to the ‘partitioning around medoids’ algorithm and ‘Flexible Procedures for Clustering’ R library and multiple database sources, including BioPlanet, REACTOME, WikiPathways, and NCBI. The heatmap and volcano plot revealed that the mice treated with DSS after C:15 supplementation for three weeks had altered gene expression in comparison with the control mice (Figure 5A,B). The heatmap shows that the expression of 20 genes was significantly altered, out of which 16 were upregulated, and 4 genes were downregulated. The altered genes of interest were involved in different immune pathways related to cytokines and inflammatory response (IL1, IL6, IL11, IL15, IL1R1, IL1RL1, and CSF3), matrix metalloproteinases (MMP3, MMP9, MMP14, and TIMP1), and collagen and extracellular matrix degradation (MMP3, MMP9, and MMP14).

## 4. Discussion

Nutritional interventions have demonstrated efficacy in ameliorating clinical symptoms and reducing inflammation in IBD patients [32]. As the complex relationship between diet and inflammation continues to evolve, an increasing number of reports support the anti-inflammatory role of some fatty acids [33] but not others [34]. For example, oleic acid, a monounsaturated fatty acid found abundantly in olive oil and certain nuts, has demonstrated anti-inflammatory properties [33,35]. However, arachidonic acid, a polyunsaturated omega-6 fatty acid prevalent in animal-derived foods like meat and eggs, has been associated with enhancing inflammation and aggravating symptoms of IBD [35]. In another study, eicosapentaenoic acid (EPA) significantly ameliorated DSS-induced colitis, alleviated the adverse effects such as weight loss, and enhanced the overall health of animals [36]. The potential of odd-chain fatty acids, like pentadecanoic acid (C:15), in relation to IBD remains unexplored. Understanding the role of different fatty acids in experimental IBD could offer insights for future therapies. Hence, we investigated the potential anti-inflammatory properties of pentadecanoic acid, an odd chain fatty acid (OCFA), in SPF-SAMP mice. Our research revealed that C:15 exhibits anti-inflammatory effects in both DSS colitis and chronic ileitis in SPF-SAMP mice.

In this study, we show that C:15 administration for 3 weeks prior to DSS induction elicits a protective effect against colitis severity, specifically protecting mice against weight loss and resulting in lower fecal MPO activity when compared to non-treated mice. Reduced fecal MPO activity is indicative of decreased neutrophil infiltration, which is a hallmark of inflammation. In a previous study, the anti-inflammatory effects of a fatty acid derivative, 17S-epoxy-docosapentaenoic acid, were reported both in human macrophage cells induced by LPS and in a mouse model of UC induced by DSS [37]. C:15 also aided in the preservation of intestinal gut integrity, as evidenced by reduced FITC and colonoscopy scores, suggesting that C:15 has protective effects on the structural integrity of the gut wall. It has been previously reported that various fatty acids, such as eicosapentaenoic acid (EPA) and docosahexaenoic acid (DHA), play a major role in the inhibition of neutrophil infiltration and help in maintaining gut integrity by protecting various tight junction proteins [38]. Our findings are in line with previous studies on different fatty acids. Specifically, a previous study reported that the supplementation of diet with omega-3 fatty acids exhibits a mitigating effect in experimental UC [39]. Similarly, in another study, the supplementation of fish oil rich in omega-3 fatty acids maintained intestinal integrity and protected against UC-related inflammation without any toxicity [40]. The anti-oxidative and anti-inflammatory properties of EPA and DHA were also reported in a previous study in which the dietary intervention of both protected against oxidative stress and intestinal mucosal injury [41].

The NanoString analysis revealed that the C:15 supplementation, along with reducing the severity of intestinal inflammation, had an impact on the expression of different genes associated with immunological pathways related to the signaling of cytokines and inflammatory response, activation of matrix metalloproteinases, collagen, and extracellular matrix remodeling in mice. The genes related to cytokines and inflammatory response play a complex role in IBD and inflammatory-related disorders, with both pro- and anti-inflammatory functions. The expression of IL-6 was increased in the mice supplemented with C:15. In a previous study, IL-6 deficiency promoted the development of colitis by recruiting more neutrophils, which secreted MPO and destroyed the intestinal epithelium [42]. IL-11, a member of the IL-6 family of cytokines, is produced by inflammatory fibroblasts during acute colitis. However, the expression of IL-11 was increased in the mice treated with C:15 as compared to the control. The results coincided with a previous study that showed that IL-11 enhanced the survival and growth of colon epithelial cells and ameliorated DSS-induced acute colitis in mouse models [43]. Similarly, the expression of genes related to IL-1 signaling (i.e., IL-1R1 and IL-1RL1) was also increased in the mice treated with C:15. There are reports that suggest that both IL-1R1 and IL-1RL1 are important in the immune network required for the restoration of epithelial barrier function in experimentally inflamed colons [44]. The expression of IL-15 was significantly decreased in C:15-treated mice. Of note, previous reports show that IL-15 has a pro-inflammatory role and is highly expressed in IBD patients, as well as promotes intestinal dysbiosis and increases susceptibility to colitis [45,46]. Cytokines are essential in IBD as they regulate the inflammatory response and activate the adaptive immune system. Their levels and expression profiles can vary depending on the disease stage and the individual patient [47]. Further, the timing and localization of cytokine levels guide the onset, recurrence, and worsening of inflammation in IBD. Therefore, the differential regulation of cytokines highlights the epithelium healing and barrier function by C:15, suggesting it is a key therapeutic agent in IBD [48]. The expression of CSF3, an essential component of the intestinal response to prevent colitis and restore mucosal barrier integrity [49], was highly expressed in the C:15-treated mice. Similarly, the expression of genes related to collagen degradation and matrix metalloproteinases (TIMP1, MMP3, MMP9, and MMP14) were also increased in the mice treated with C:15. Naoki et al., 2019 reported that the production of TIMP-1 by the monocytes in the colon inhibits collagen degradation [50]. Various studies have reported that the expression of various MMPs has both anti-inflammatory and pro-inflammatory effects [51,52,53]. In particular, MMP-3 has been found to have anti-inflammatory effects, wherein MMP-3 treatment decreased the number of macrophage and antigen-presenting cells and significantly inhibited pro-inflammatory cytokines, while the inhibition of MMP-3 activity eradicated these anti-inflammatory effects [51]. In another study, MMP-9 overexpression inhibited pro-inflammatory cytokine production and helped to protect against LPS-induced inflammation [52]. Similarly, in another study, MMP-14 was found to trigger an anti-inflammatory proteolytic cascade, such as activation of MMP-2, which helps in decreasing inflammation [53]. The expression of TLR-2 and NOS-2 both decreased in the mice treated with C:15. Previous studies have shown that TLR-2 and NOS-2 expression decreases with anti-inflammatory therapies and that the dysregulation or deletion of these genes is crucial for both IBD pathogenesis and attenuation of DSS induced colitis severity in mice [54,55,56,57]. The expression of H2-Q1 was also decreased in C:15 treated mice; however, at present, no studies have identified the relevance of the gene to inflammation in relation to IBD or colitis. The expression of OSMR was increased in the C:15-treated mice, which is in accordance with the previous study showing the expression of OSMR in healthy and non-inflamed mouse tissue [58]. The expression of HGF, CD163, NFIL3, ITGB8, and LPL, all crucial mediators of inflammation, was increased in the mice treated with C:15. HGF has been shown to play a role in attenuating inflammation [59,60]. Similarly, several studies have shown the anti-inflammatory role of CD163 in the reduction of inflammation and oxidative stress [61,62], while NFIL3 deficiency promotes inflammation [63]. The ITGB8-mediated immune pathway is essential for preventing immune dysfunction, autoimmunity, and IBD [64]. LPL can limit inflammation by generating natural PPARa ligands from the hydrolysis of triglyceride (TG)-rich lipoproteins [65]. Previous studies suggest that the expression of PTSG-2 is increased after DSS treatment [66]. However, C:15 did not control the expression of PTSG-2 in our study, which might be due to the complexity of the binding site of PTSG-2 [67]. The changes in cytokine expression can positively or negatively affect the integrity of the intestinal epithelial barrier by regulating epithelial cell functions such as proliferation, apoptosis, and permeability [68,69]. Further, the intestinal wall is a dynamic network maintained by the epithelium and ECM, which renews constantly under healthy physiological conditions. However, during tissue damage, metalloproteinases play an important role in maintaining gut integrity by participating in tissue remodeling and homeostasis. They are expressed in all layers of the intestinal wall by different cell types and are involved in processes such as angiogenesis, cell migration, tissue repair, and inflammation [70,71]. These findings align with existing research suggesting the role of various genes involved in inflammatory response, ECM remodeling, collagen regeneration, and matrix metalloproteinases as potential therapeutic targets in IBD [72,73,74].

Overall, our findings demonstrate that C:15 has significant anti-inflammatory potential, protecting against colitis by reducing weight loss, fecal MPO activity, and less translocation of FITC dextran, indicating a decrease in inflammation and preserving gut integrity. Gene expression analysis revealed that C:15 altered the expression of various genes related to multiple immunological and tissue remodeling pathways, including the modulation of cytokines, matrix metalloproteinases, and ECM components. These results suggest that C:15 may offer a promising avenue for future IBD therapies, contributing to a broader understanding of the complex relationship between diet, fatty acids, and inflammation.

## 5. Conclusions and Future Directions

This study suggests that pentadecanoic acid has significant anti-inflammatory potential that effectively decreases the severity of colitis by reducing inflammation and maintaining the integrity of the gut in different mouse models. C:15’s effect on immune response and tissue remodeling genes underlines its potential as a therapeutic agent for IBD, contributing to a broader understanding of the complex relationship between diet, fatty acids, and inflammation in relation to IBD.

While our research provides valuable insight into the anti-inflammatory properties of C:15, it is important to recognize certain limitations. First, this study was carried out using mice models, limiting the direct application of these findings to clinical settings. Furthermore, this study concentrated on gene expression associated with immunological responses and tissue remodeling but did not thoroughly investigate the mechanism of action and the molecular pathways that could be impacted by C:15. Further study is required to elucidate the molecular mechanism and pathways by which C:15 affects inflammation and gut integrity, as well as to validate these findings in human clinical trials. The dietary intervention in humans will be focused on exploring the optimal dosage, delivery, safety, and influence of dietary interactions for personalized C:15 supplementation to IBD patients. Additionally, investigating the efficacy of C:15 along with other anti-inflammatory compounds or dietary agents to evaluate any synergistic effects could be helpful in tailoring its use for IBD therapy.

## Figures and Tables

**Table 1:** Effect of C:15 on different parameters among the various mouse models.

Experiment	DSS Induced Colitis	DSS Induced Colitis	Chronic Ileitis
C:15 administration	Short term (3 weeks)	Long term (6 weeks)	6 weeks
Mouse (age)	SAMP/YitFc (14 weeks)	SAMP/YitFc (14 weeks)	SAMP/YitFc (24 weeks)
Comparison	C:15(1X) vs. Control	C:15(1X) vs. (2X) vs. Control	C:15(1X) vs. Control
% Residual Body weight *	C:15: 103.1 ± 2.0 Control: 92.0 ± 2.8	C:15(1X): 97.5 ± 2.9 C:15(2X): 99.5 ± 2.5 Control: 97.1 ± 1.7	C:15: 105.2 ± 1.4 Control: 96.3 ± 1.3
MPO U/mg Log_2_ *	C:15: 2.5 ± 0.5 Control: 5.2 ± 0.6	C:15(1X): 3.1 ± 0.2 C:15(2X): 3.2 ± 0.9 Control: 7.1 ± 0.1	C:15: 4.2 ± 0.5 Control: 6.2 ± 0.9
Fluorescence Log_2_ *	C:15 18.6 ± 0.7 Control: 21.4 ± 0.6	C:15(1X): 19.0 ± 2.1 C:15(2X): 17.6 ± 0.5 Control: 24.0 ± 0.5	C:15: 15.4 ± 0.2 Control: 15.8 ± 0.2
Colon length *	C:15: 7.0 ± 0.6 Control: 5.6 ± 0.4	C:15(1X): 6.5 ± 0.4 C:15(2X): 6.3 ± 0.5 Control: 5.6 ± 0.3	NA
Colonoscopy *	C:15: 3.1 ± 1.0 Control: 4.4 ± 1.5	C:15(1X): 1.6 ± 0.8 C:15(2X): 1.6 ± 0.8 Control: 4.7 ± 0.9	NA
Colon Histology *	C:15: 11.0 ± 4.3 Control: 17.8 ± 1.6	C:15(1X): 10.9 ± 4.8 C:15(2X): 6.3 ± 2.3 Control: 8.9 ± 3.4	NA

* All the values presented in the table are from the terminal day of the experiments. Values are Mean ± SD.

## Data Availability

The original contributions presented in the study are included in the article/supplementary material; further inquiries can be directed to the corresponding author.

## Supplementary Materials

The following supporting information can be downloaded at: https://www.mdpi.com/article/10.3390/nu16173031/s1, Figure S1: C:15 given for 3 weeks reduces the severity of acute chemical colitis in 14 wk old SPF SAMP.
