# Supplementary material for: Anti-Inflammatory Effect of Dietary Pentadecanoic Fatty Acid Supplementation on Inflammatory Bowel Disease in SAMP1/YitFc Mice
Source: Nutrients. 2024 Sep 8;16(17):3031. doi: 10.3390/nu16173031 (PMC11397537; doi:10.3390/nu16173031)
Supplement: Supplementary file 1 [file nutrients-16-03031-s001.zip › nutrients-3196277-supplementary.pdf]

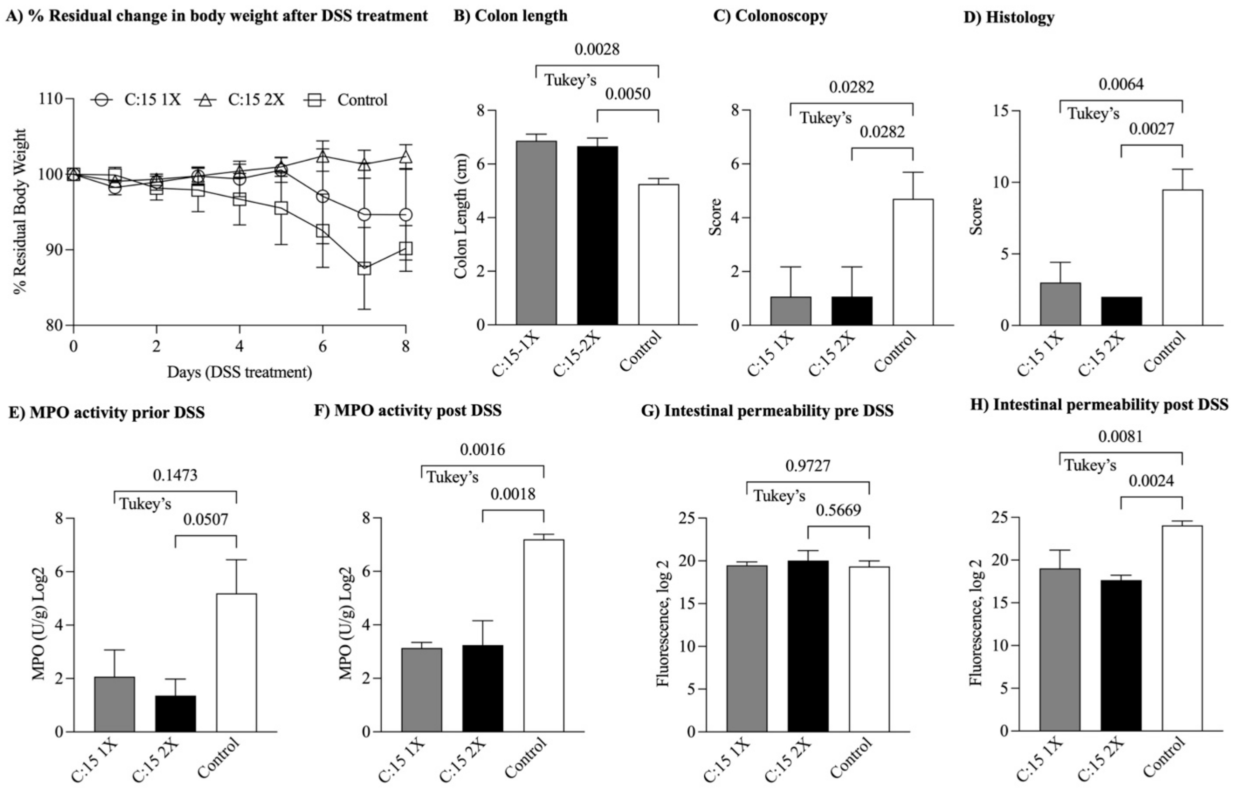

Figure S1. C:15 given for 3 weeks reduces the severity of acute chemical colitis in 14-wk-old SPF SAMP. Values are mean ± SD, *n* = 3. (**A**) percentage change from original body weight (defined as day 0 and as 100%) after induction of DSS-colitis, (**B**) colon length (in cm), (**C**) colonoscopy score, (**D**) colon histology scores, (**E**) fecal MPO activity before DSS and (**F**) after DSS treatment, (**G**) FITC-Dextran before DSS, and (**H**) after DSS treatment, (FITC, fluorescein isothiocyanate; MPO, myeloperoxidase; KW- Kruskal–Wallis ANOVA; ANOVA- Analysis of Variance).
